# Trypanothione Reductase: A Target Protein for a Combined *In Vitro* and *In Silico* Screening Approach

**DOI:** 10.1371/journal.pntd.0003773

**Published:** 2015-06-04

**Authors:** Mathias Beig, Frank Oellien, Linnéa Garoff, Sandra Noack, R. Luise Krauth-Siegel, Paul M. Selzer

**Affiliations:** 1 MSD Animal Health Innovation GmbH, Zur Propstei, Schwabenheim, Germany; 2 Universität Heidelberg, Biochemie-Zentrum (BZH), Heidelberg, Germany; 3 Universität Tübingen, Interfakultäres Institut für Biochemie, Tübingen, Germany; 4 Wellcome Trust Centre for Molecular Parasitology, Division of Infection, Immunity and Inflammation, Faculty of Biomedical & Life Sciences, University of Glasgow, Glasgow, United Kingdom; Northeastern University, UNITED STATES

## Abstract

With the goal to identify novel trypanothione reductase (TR) inhibitors, we performed a combination of *in vitro* and *in silico* screening approaches. Starting from a highly diverse compound set of 2,816 compounds, 21 novel TR inhibiting compounds could be identified in the initial *in vitro* screening campaign against *T*. *cruzi* TR. All 21 *in vitro* hits were used in a subsequent similarity search-based *in silico* screening on a database containing 200,000 physically available compounds. The similarity search resulted in a data set containing 1,204 potential TR inhibitors, which was subjected to a second *in vitro* screening campaign leading to 61 additional active compounds. This corresponds to an approximately 10-fold enrichment compared to the initial pure *in vitro* screening. In total, 82 novel TR inhibitors with activities down to the nM range could be identified proving the validity of our combined *in vitro/in silico* approach. Moreover, the four most active compounds, showing IC_50_ values of <1 μM, were selected for determining the inhibitor constant. In first on parasites assays, three compounds inhibited the proliferation of bloodstream *T*. *brucei* cell line 449 with EC_50_ values down to 2 μM.

## Author Summary

Trypanosomatidae are responsible for approximately half a million human fatalities per annum and the situation is compounded by substantial economic losses due to affecting live stock as well. Trypanothione reductase (TR) is an essential key enzyme of the unique trypanothione-based thiol metabolism of the trypanosomatidae and TR is a promising target for the development of selective inhibitors. However, TR is a very hard to attack target in standard drug discovery approaches. Therefore, we developed a combined and iterative *in vitro* and *in silico* screening approach, which led to a high number of novel TR inhibitors. 82 of those showed activities down to the nM range against *T*. *cruzi* TR. Moreover, the four most active compounds were selected for determining the inhibitor constant. In first on parasite efficacy studies, three of those compounds inhibited the proliferation of bloodstream *T*. *brucei* cell line 449 with EC_50_ values down to 2 μM.

## Introduction

Trypanosomatidae are responsible for approximately half a million of human fatalities per annum in subtropical and tropical regions around the world [1]. *Trypanosoma brucei rhodesiense* and *T*. *b*. *gambiense* are the causative agents of African sleeping sickness [2]. *T*. *cruzi* is responsible for Chagas’ disease. The disease complex Leishmaniasis including Kala Azar (*Leishmania donovani*) is caused by different species of *Leishmania*. Above all, these parasitic protozoa cause substantial economic losses by affecting life stock (*T*. *congolese*, *T*. *b*. *brucei*, *T*. *evansi*) [3], [4]. For the treatment of the diseases only a handful of chemotherapeutics are available and their efficacy suffers from widespread drug resistance and serious side effects. Thus, there is an urgent need to discover new compounds as starting point for the development of potent drugs with less side effects, preferably interfering with unique essential pathways of these parasites [5], [6].

**Fig 1:**
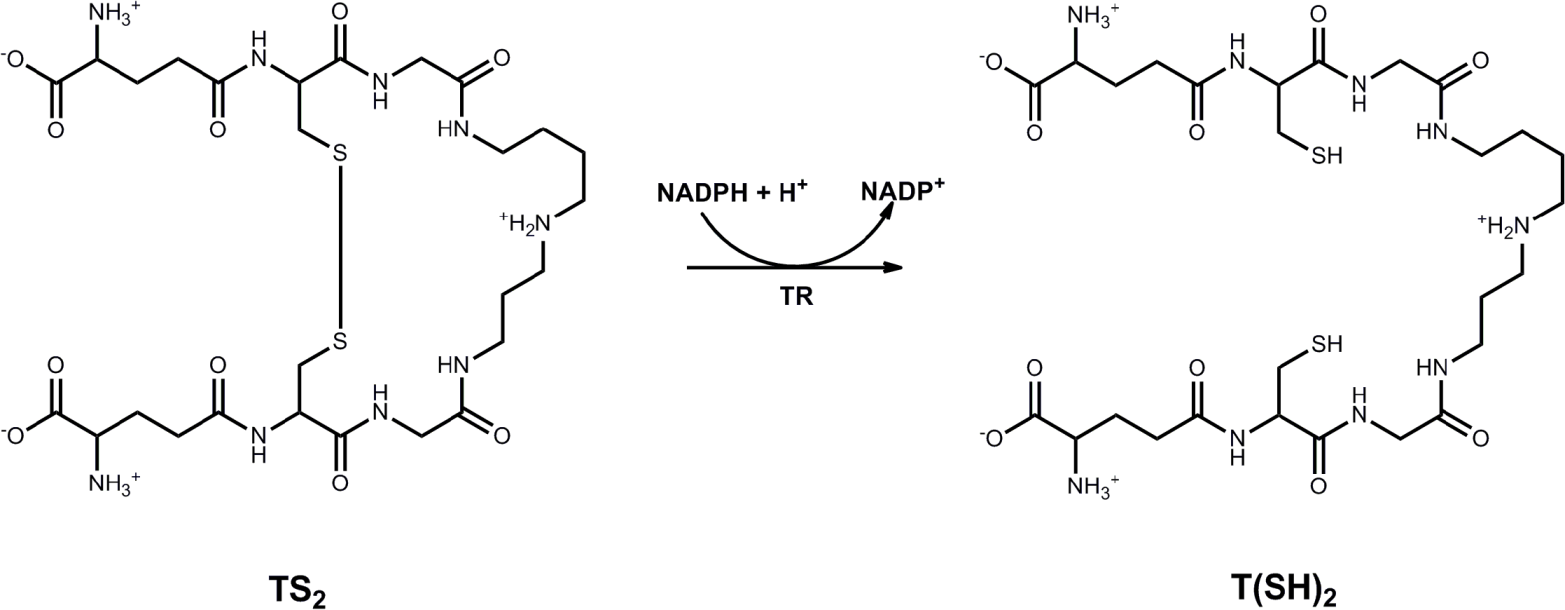
TR-catalyzed reduction of trypanothione disulfide (TS_2_) to the dithiol trypanothione (T(SH)_2_).

Trypanothione reductase (TR) is an essential enzyme of the unique trypanothione-based thiol metabolism of Trypanosomatidae [7], [8]. The flavoenzyme catalyzes the NADPH-dependent reduction of trypanothione disulfide [TS_2_] to the dithiol trypanothione [bis(glutathionyl)spermidine, T(SH)_2_] (Fig 1) [1], [9], [10]. Trypanosomatids lack both glutathione reductase (GR) and thioredoxin reductase and therefore TR is the only connection between the NADPH- and thiol-based redox systems [11], [12]. T(SH)_2_ is the substitute for many pathways and antioxidant functions [13], [14], [15] which in other organisms including the mammalian host are fulfilled by the glutathione and/or thioredoxin systems. Parasites with reduced TR levels are highly sensitive towards oxidative stress [7]. The nearest homologue of TR in human cells is GR with about 40% sequence identity. However, both enzymes display significant differences with respect to their active sites which results in a mutually exclusive specificity towards their disulfide substrate. TR was validated by different genetic approaches to be essential for the proliferation of *Leishmania* and *Trypanosoma* [7], [8], [16]. Taken together, these facts render TR a promising target for the development of selective inhibitors.

**Fig 2:**
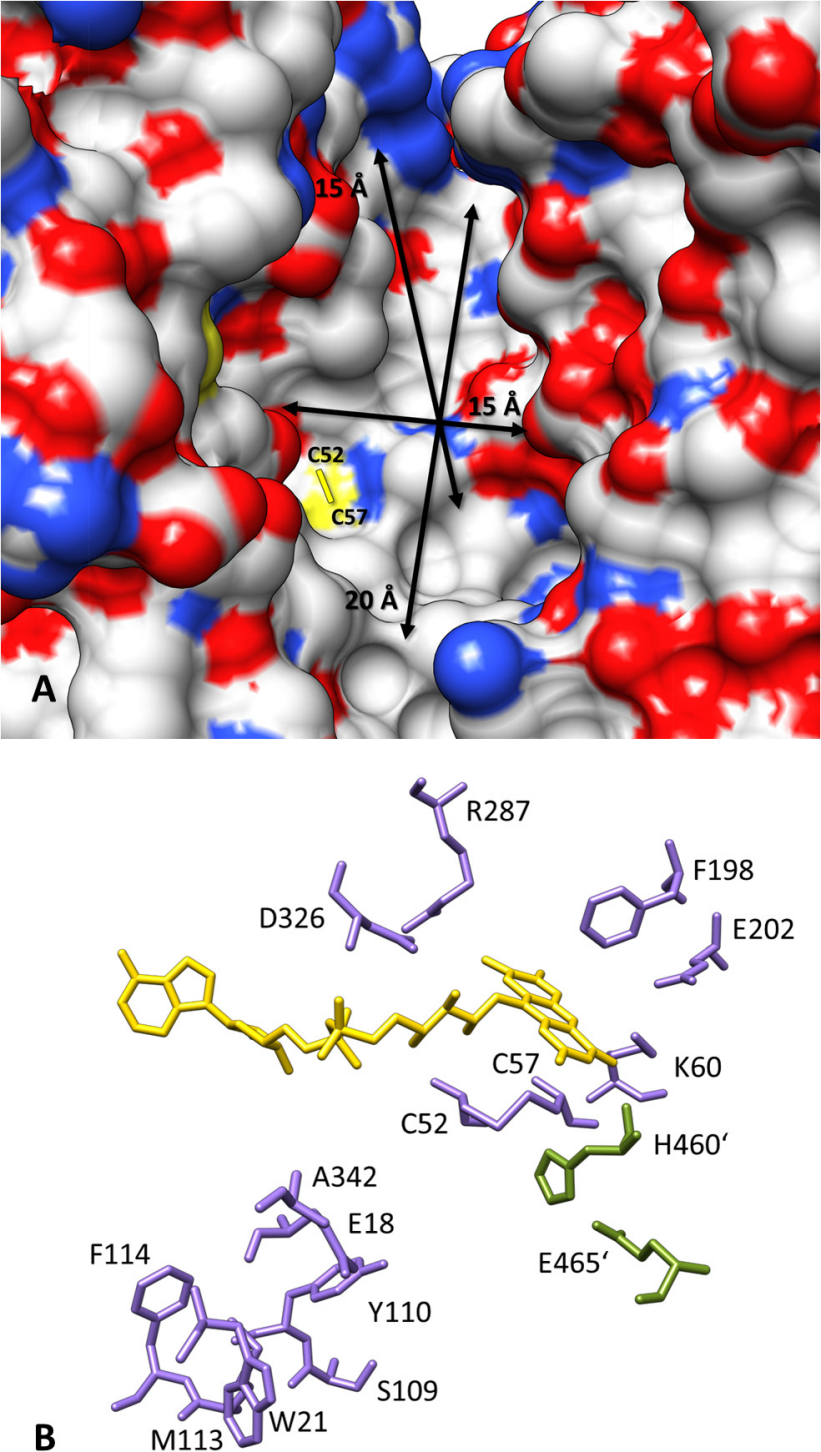
Schematic presentation of the active site of *T*. *cruzi* TR. (A) Top view into the large trypanothione disulfide binding site having a dimension of about 15 x 15 x 20 Å (arrows). The disulfide bridge formed by C52 and C57 in the oxidized form of the enzyme is indicated. The cofactor FAD is not visible because it is buried within the structure. On the solvent accessible surface nitrogen is indicated as blue, oxygen as red and sulfur in yellow. (B) The isoalloxazine ring of FAD (yellow) forms the center of the active site. NADPH binds at the re-site, while TS_**2**_ binds at the si-site of the flavin ring where also the redox active dithiol/disulfide couple of Cys52-Cys57 is located. Glu18 (Ala34 in human GR), Trp21 (Arg37), Ser109 (Ile113), Met113 (Asn117) and Ala342 (Arg347) are the five residues in the active site that are not conserved when comparing TR with human GR. Primed residues (green) are provided by the second subunit of the homodimeric protein. The substitution of Ala34 and Arg37 into Glu and Trp, respectively, converts human GR in an enzyme with TR activity and vice versa.

A typical target-based approach starts either with high throughput screening of large libraries of small molecules [17] or with *in silico* experiments like a virtual screening to create a focused data set containing *in silico* hits which are subsequently tested by *in vitro* assays [18], [19], reducing screening costs significantly. Although several crystal structures of TR are available, their applicability for common structure-based virtual screening campaigns is inappropriate compared to other druggable protein targets like proteases [20], [21], [22], [23] or kinases [24], [25], [26]. TR has a very wide and featureless active site with approximate dimensions of 15 x 15 x 20 Å (Fig 2) [27], [28]. In addition, the mainly hydrophobic TS_2_ binding site does not provide many directed interactions like hydrogen bonds. Therefore, ligands can bind with many different binding modes all over the active site. As a consequence, *in silico* approaches like molecular docking or pharmacophore screening are not capable to identify a reasonable and correct binding conformation [19], [21], [27].

Here we describe the approach that started with an *in vitro* screening of a highly diverse compound library to come up with a set of hits. These compounds were then used as a starting point for a ligand-based *in silico* screening that resulted in a focused library of similar structures with potential TR activity. Finally, the activity-enriched data set was subjected to a second *in vitro* screening campaign. This iterative combination of *in vitro* and *in silico* screening methods led to a higher number of TR inhibitors compared to pure *in vitro* or *in silico* approaches.

## Material and Methods

The compounds used in the enzymatic inhibitory testing and the *in silico* screening were available as stock solutions in DMSO or in solid from a compound library maintained at MSD. All IC_50_ determinations were performed starting from solid material. Recombinant *T*. *cruzi* TR (CL strain) was prepared using a published expression system [29]. TS_2_ was prepared enzymatically as described previously [30]. For antiparasitic studies, culture-adapted bloodstream *T*. *brucei* parasites of cell line 449 were used [31]. These cells are descendants of strain Lister 427 stably transfected with the gene for the tetracycline repressor.

### Enzymatic assay

The kinetic analysis of TR was performed in 384-well plates (Greiner Bio-One GmbH, Frickenhausen, Germany). The reaction mixture (50 μl) contained 5 mU/ml TR, 40 mM Hepes, pH 7.5, 1 mM EDTA, 300 μM NADPH (Sigma-Aldrich), 0.1 mg/ml BSA, and 0.01% Pluronic (BASF). The detergent was used to prevent the formation of droplets during pipetting and had no negative effects on the assay kinetics. In a volume of 30 μl, the assay components and the inhibitor were pre-incubated for 30 minutes and the reaction started by adding 20 μl of 375 μM TS_2_ resulting in final concentrations of 150 μM TS_2_ and 2% DMSO. The absorption decrease at 340 nm was measured by start and end point determination resulting in a delta of the optical density (delta A) for 30 min. All experiments were performed at room temperature. The TR concentration was kept constant at 5 mU/ml for all types of experiments. Therefore, after an initial calculation of the turnover number (k_cat_), only delta A values under identical conditions were compared. Initial screening was performed twice in two independent experiments.

### IC_50_ determination

Eleven compound concentrations were used ranging from 100–0.01 μM or 200–0.004 μM. The compounds were freshly dissolved in DMSO. All measurements were performed twice in three independent series. Enzyme activities were plotted versus increasing inhibitor concentrations. IC_50_ values were calculated using the four-parameter equation model 205 and the option “unlock” from the XLfit add-in (IDBS, Guildford, United Kingdom) in Excel (Microsoft Corporation, Redmond, WA).

### *In silico* similarity search

The similarity search workflow was implemented by using the workflow application Pipeline Pilot [32]. TGT, TGD and MACCS fingerprints [33] were calculated by the MOE program [34] while ECFP6 and FCFP4 fingerprints were provided by built-in Pipeline Pilot components [32]. TGD and TGT fingerprints are pharmacophore-based descriptors. While TGD represents the existence of topological binding property pairs for seven pre-defined pharmacophore features, TGT encodes triplets of pharmacophore features for four pre-defined pharmacophore features. MACCS fingerprints are substructure descriptors encoding the presence of up to 960 molecular patterns. Extended Connectivity Fingerprints (ECFP) and Functional Connectivity Fingerprints (FCFP) are topological descriptors encoding information on atom-centered fragments. Within the workflow all six fingerprints for each of the 21 active query structures were calculated resulting in 126 (21 x 6) single similarity searches. Each similarity search was limited to a result set containing the 13 most similar hits. Afterwards the resulting structures were combined and duplicates retrieved by different similarity searches were removed.

All *in silico* hits passing the *in vitro* hit confirmation step were filtered towards the existence of Pan Assay Interference compounds (PAINS) [35]. The filter was implemented by using the 480 defined PAINS substructures and the ‘Substructure Map’ component of Pipeline Pilot [32].

### Mode of inhibition studies

Before determining kinetic constants reversibility of compound binding has to be tested. Therefore, Amicon Ultra 0.5 ml centrifugal filters with a cut-off of 10,000 MW (Millipore Corporation, Billerica, MA, USA) were washed with 400 μl TR assay buffer and centrifuged for 5 min at 12,500 rpm at room temperature. Remaining buffer was removed by placing the filter upside down into a microcentrifuge tube and centrifugation at 3,500 rpm for 2 min. The filter was then loaded with 50 μl assay mixture containing 5 mU/ml TR (50 kDa), 100 μM inhibitor and 300 μM NADPH or the control mixtures which had been incubated for 30 min. The filter was centrifuged for 15 min at 12,500 rpm, washed three times with 400 μl buffer and centrifuged again. The retentate was collected by placing the filter upside down on a new tube and centrifugation at 3,500 rpm for 2 min. The recovered protein solutions were subjected to a standard TR assay.

### K_i_ determination

Measurements were performed at 60, 80, 120, and 200 μM TS_2_ and five inhibitor concentrations resulting in 20 different data points per inhibitor. The K_i_ values were derived from two independent experiments each done in duplicate. SigmaPlot 10 (Systat Software, Inc., San Jose, USA) with the Enzyme Kinetics Module was used to evaluate the inhibition type and K_i_ values. This software automatically estimates the initial parameters for different fit models, and uses the Marquardt-Levenberg algorithm to determine the parameter values. A detailed statistical report as well as a data report is generated to compare multiple models and graphs. Based on these statistics, the appropriate binding model can be selected. Graphs are created based on the models and the calculated parameter values instead of fitting each individual inhibitor concentration curve.

### *In silico* ADME parameters and physicochemical properties

ADME parameters and physicochemical properties were calculated and predicted for the four nanomolar inhibitors and chlorhexidine by using the software packages Pipeline Pilot by Accelrys [32] and Volsurf+ by Molecular Discovery [36]. The measured properties cover standard descriptors like number of H-bond donors and acceptors, number of rotatable bonds, molecular weight, logP and PSA but also ADME features like permeability (logBB [36], Blood Brain Barrier Leve l [32], SKIN [36], CACO2 [36]), solubility (Molecular Solubility [32], logS7.5 [36], ADME Solubility Level [32]), metabolic stability [36], protein binding [32] and intestinal absorption [32].

### Cell culture activity determination by counting living cells

Compounds were dissolved in DMSO to 10 mM stock solutions and added to bloodstream *T*. *brucei* (strain 449) in concentrations of 100, 50, 5, 0.5, and 0.05 μM in 24-well plates. The final DMSO content in the cultures was 1, 0.5, 0.05, 0.005 and 0.0005%, respectively. The initial cell density was 2500 cells/ml. After 48 h and 72 h incubation at 37°C in HMI-9 medium, viable cells were counted in a Neubauer chamber. The assay was performed in triplicate. The parasite strain and the culture conditions are described in the literature [31].

### EC_50_ determination by ATPlite 1step assay system

The ATPlite 1step assay system (PerkinElmer, Waltham, MA, USA) was used as described in Füller *et al*. [37] to quantify *T*. *brucei* survival in medium-throughput dose-response series. Experiments were conducted in 96-well microplates (PerkinElmer, Waltham, MA, USA), each well containing 90 μl cell-culture and 10 μl compound. Initial cell densities were 2500 cells/ml. Compounds 1 and 2 were diluted stepwise with HMI-9 medium from the 10 mM DMSO stock solutions. Unfortunately, compound 3 was no longer available for testing. The highest concentration of DMSO added with the compounds was 0.5% which did not affect viability of the parasites. Treatment with 10% DMSO served as positive control (100% inhibition). For each compound, three identical plates were prepared, incubated at 37°C and analyzed after 24 h, 48 h, and 72 h, respectively. Samples from each cell-line left untreated were added to the plates prior to each measurement as additional controls. 50 μl of the ATPlite 1step solution (PerkinElmer, Waltham, MA, USA) were added to each well and the relative luminescence was measured immediately using a VICTOR Multilabel Plate Reader (PerkinElmer, Waltham, MA, USA) at room temperature. The values obtained were plotted against the logarithmic compound concentrations. A dose-response curve was generated from which EC_50_-values were calculated using the program PRISM 5.0 (GraphPad Software, La Jolla, CA, USA).

### Compound quality control data

#### Compound 1

^1^H-NMR (DMSO) *δ* 3.75 (2H, t, J = 6.00 Hz), 4.47 (2H, t, J = 6.00 Hz), 7.00 (1H, t, J = 7.37 Hz), 7.27 2H, t, J = 7.80 Hz), 7.44 (2H, d, J = 7.89 Hz), 7.67 (1H, d, J = 8.01 Hz), 8.57 (1H, d, J = 7.95 Hz), 9.72 (1H, s). HPLC-MS, [M+H]^+^ 361, C_15_H_12_N_4_O_5_S, Purity at 254 nm 78,3%, Purity at 210 nm 100%.

#### Compound 2

^1^H-NMR (DMSO) *δ* 7.88 (1H, t, J = 7.37 Hz), 8.11 (2H, m, J = 7.08 Hz), 8.34 (1H, d, J = 8.28 Hz), 8.48 (1H, s), 8.95 (1H, s). HPLC-MS, [M+H]^+^ 376, Purity at 254 nm 87%, Purity at 210 nm 100%.

#### Compound 3

^1^H-NMR (DMSO) *δ* 8,41 (1H, s). HPLC-MS, Purity at 254 nm 94,5%, Purity at 210 nm 100%.

#### Compound 4

^1^H-NMR (DMSO) *δ* 7.46 (1H, s), 7.51 (2H, s), 7.65 (2H, d, J = 8.37 Hz), 7.97 (2H, d, J = 8.37 Hz), 11.76 (1H, s). HPLC-MS, [M+H]^+^ 370, Purity at 254 nm 100%, Purity at 210 nm 100%.

## Results and Discussion

### Assay adaption and evaluation

**Fig 3:**
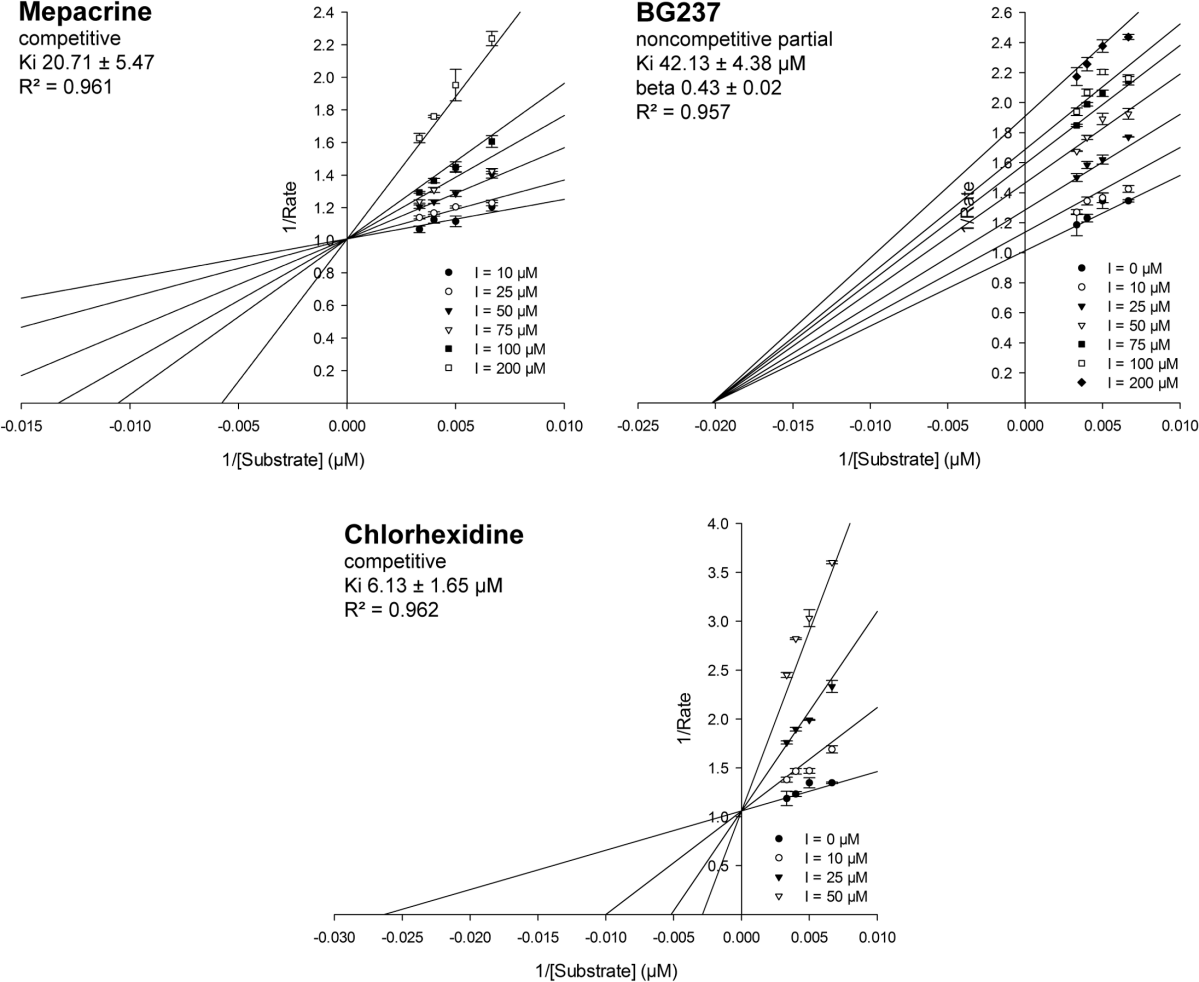
TR assay validation—Lineweaver-Burk plots for known inhibitors. The K_**i**_ values were determined by three independent experiments. **Mepacrine:** measured Ki 20.71 ± 5.47 μM, competitive binding mode; **BG237:** measured Ki 42.13 ± 4.38 μM, noncompetitive binding mode, partial. The factor beta reflects the modification of the rate of product formation by the enzyme that is caused by the inhibitor. **Chlorhexidine:** measured Ki 6.13 ± 1.65 μM, competitive binding mode. Graphs are created using the SigmaPlot Enzyme Kinetics Module routine based on the relevant binding models and the calculated parameter values.

For the initial *in vitro* screening a robust assay was developed based on recombinant *T*. *cruzi* TR, which is much more stable than the recombinant *T*. *brucei* enzyme. This is reasonable since both proteins display a sequence identity of more than 80% and show comparable inhibition [38]. A robust NADPH-linked photometric assay [39] was chosen that firstly had to be adapted to the high throughput screening format. The assay volume was reduced from the 1 ml cuvette format to 50 μl total volume in 384 well plates. A high concentration of TS_2_ was chosen to mimic severe oxidative stress conditions and to discriminate against weak competitive inhibitors. In order to stabilize the enzyme and to prevent adhesion and reduce surface tension as well as the formation of droplets in the small wells, 0.1 mg/ml BSA and 0.01% detergent (Pluronic) were added to the reaction mixture. The assay was validated by a) comparing the published and measured K_m_ value for TS_2_ (measured K_m_ 20 μM, published K_m_ 18 μM [39] and 29.6 μM [40]) and b) comparing the measured inhibitor constants (K_i_) of three known inhibitors—chlorhexidine, mepacrine, BG237—with published data [41], [42], [43] (Table 1, Fig 3). The kinetic values obtained in the high throughput screening (HTS) assays were in the same order of magnitude as the published data. Subsequently, the IC_50_ values of the three inhibitors were determined in the screening format to evaluate the robustness of the assay. In the presence of 150 μM TS_2_, mepacrine, BG237, and chlorhexidine yielded IC_50_ values of >200 μM, 98 μM, and 26 μM, respectively (Table 1). The accordance of the measured and published kinetic values as well as the capability of the assay to discriminate against weak inhibitors confirmed the successful transfer of the original TR assay to the HTS format.

**Table 1:** TR assay validation—measured versus published kinetic data.

Inhibitor	K_i_ [μM]		IC_50_ [μM]
	Published	Measured	
**Mepacrine**	19 [39]	20 ± 5	>200
**BG237**	21 [42]	19 ± 8	98 ± 20
**Chlorhexidine**	2 [43]	4 ± 1	26 ± 2

K_i_ ± SD (n = 3); IC_50_ ± SD (n = 3)

### *In vitro* screening campaign

**Fig 4:**
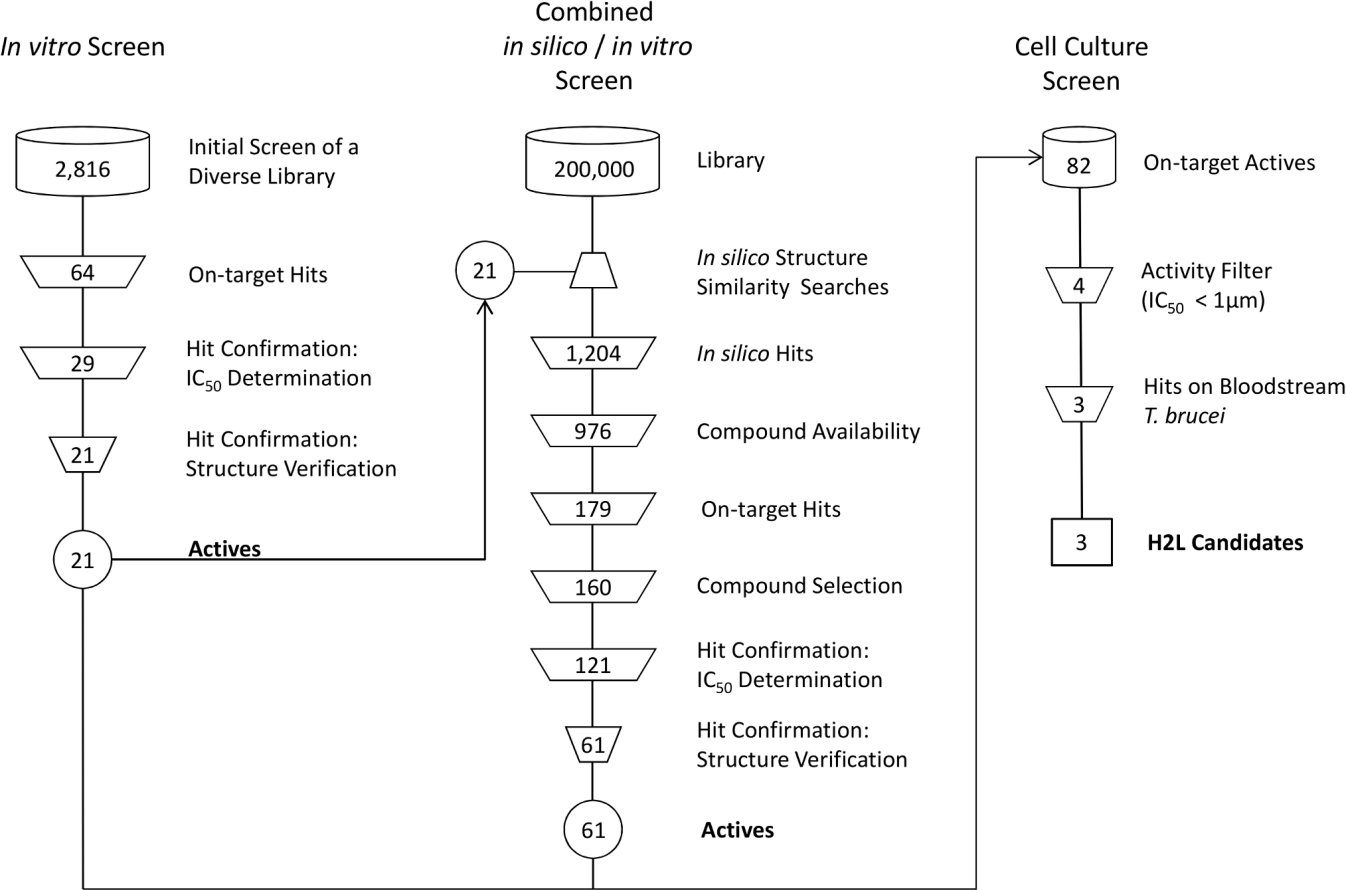
Schematic representation of the combined *in vitro* and *in silico* screening approach. After screening the highly diverse compound set of 2,816 compounds, 21 hits were obtained. These actives were then used as query for an *in silico* structure similarity search with the aim to create an activity enriched compound set. The resulting focused library of 1,204 compounds was screened again leading to additional 61 novel compounds with inhibitory activity against TR. Four out of the 82 combined, novel TR inhibitors showed activities of < 1 μM and were tested for their ability to interfere with the proliferation of cultured bloodstream *T*. *brucei*. Finally, three compounds showed activity in cell culture and were selected for further optimization efforts.

**Fig 5:**
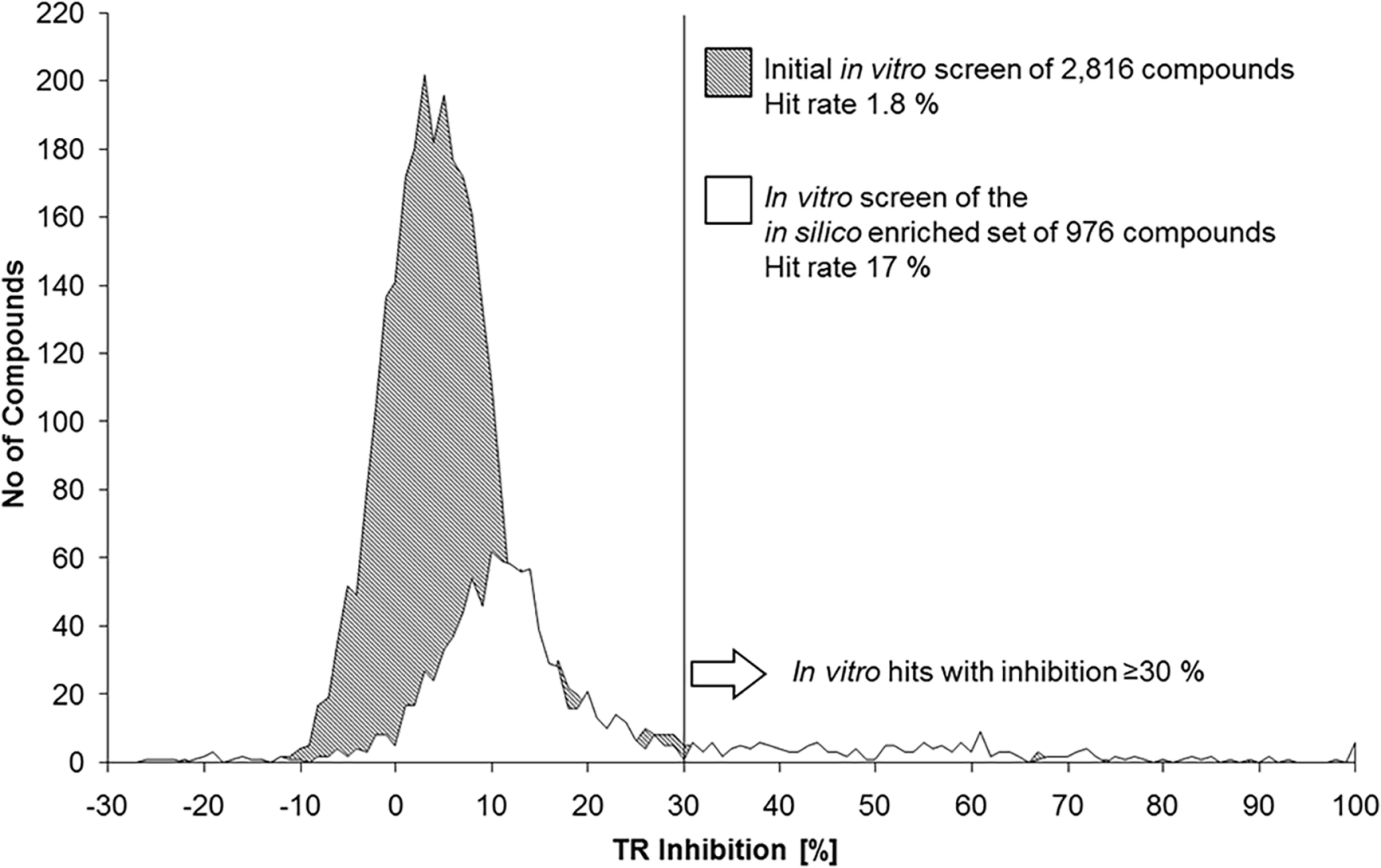
Inhibition distribution of the *in vitro* screenings. The number of compounds is plotted against the percentage of TR inhibition. The grey area represents the activity distribution of the compounds in the initial screening, while the white area shows the distribution of the second *in vitro* screening based on the *in silico* enriched focused data set. The overall activity of the *in silico* enriched data set is shifted to the right compared to the initial diverse *in vitro* screen data set. Importantly, the second screen delivered more *in vitro* hits with an inhibitory potency of ≥30% although this data set was 3 times smaller.

The initial compound library contained 2,816 chemicals that represented a highly diverse subset of the MSD screening library of over 200,000 substances. In addition, the three known inhibitors (mepacrine, BG237, chlorhexidine) used for the assay validation were included as controls. All compounds were studied at a concentration of 20 μM in the presence of 150 μM TS_2_. The screening was performed twice in two independent experiments and the data obtained were analyzed using the software ActivityBase (ID Business Solutions Ltd., Guildford, UK). The dimensionless statistical value Z-prime which assesses the measurement quality of each plate was ≥ 0.87 reflecting the robustness of the screening system [44]. The analysis showed a typical percentage inhibition distribution and 64 compounds displayed a mean inhibition of > 30% resulting in a hit rate of 1.8% (Figs 4 and 5). All 64 substances were subjected to further hit confirmation and structure verification experiments.

### *In vitro* hit confirmation

The selected 64 compounds were freshly dissolved from the solid material instead of using the stock solutions used in the primary activity screen. This procedure ensured that the observed effect was caused by the authentic compound and not by putative decomposition products that might have been formed in the stored stock solution. The determination of the IC_50_ values revealed a correlation coefficient (R^2^) of 0.968 proving that the activity of all validated compounds could be reliably determined. Finally, 29 compounds were identified that showed IC_50_ values down to 1.15 μM with 21 compounds being more potent than chlorhexidine (IC_50_ < 26 μM). After structural validation by LC/MS and NMR analyses, 21 highly active compounds could be confirmed by the initial campaign (Fig 4).

### *In silico* screening campaign

The 21 actives retrieved by the *in vitro* screening were used as new structural starting positions for a ligand-based *in silico* screening. Each *in vitro* hit was used to search for analogues in an in-house database of 200,000 compounds. Because the molecular properties responsible for the inhibitory potency were not known, six fingerprints describing and representing each structure in different ways were used instead of only one molecular descriptor. The fingerprints were selected to cover various structural aspects that might be responsible for the on-target activity like similar topology (ECFP6, FCFP_4; Accelrys [32]), similar structural fragments (MACCS Keys (public and private keys); Symyx [33]) and pharmacophoric features (TGT, TGD; CCG [34]). In summary, 126 single similarity searches were performed in parallel using the workflow program Pipeline Pilot [32]. Each search was limited to the best 13 resulting structures to ensure a high degree of similarity. Finally, a focused compound set containing 1,204 duplicate-free, novel *in silico* hits with potential TR activity was obtained.

### Screening the *in silico* hit library

976 out of the 1,204 compounds identified by the *in silico* screening campaign were available in sufficient amounts as stock solutions or solid material and could be tested in a second *in vitro* study. The compound set was studied under the same conditions as in the initial *in vitro* screening described above. Finally, 179 further hits displaying > 30% inhibition were obtained resulting in a hit rate of about 17% (Fig 5). This hit rate is nearly ten times higher compared to that in the initial screening, although the compound data set was 3-times smaller. This clearly demonstrates that the introduced *in silico* approach was able to generate a highly enriched focused library of TR inhibitors. The 160 most active hits were selected for IC_50_ measurements using 11 concentrations ranging from 200 to 0.003 μM. The activity of 121 substances could be confirmed by IC_50_ values of which most were more potent than the well-known standard chlorhexidine. Finally, a second set of additional 61 novel TR inhibitors remained after structural confirmation (NMR and LC/MS) and substructure-based filtering against Pan Assay interference compounds [35] (Fig 4). Combining all actives from the first and second *in vitro* screening campaign, 82 compounds active on TR with confirmed *in vitro* activity could be identified.

### Mode of inhibition and kinetic studies

**Fig 6:**
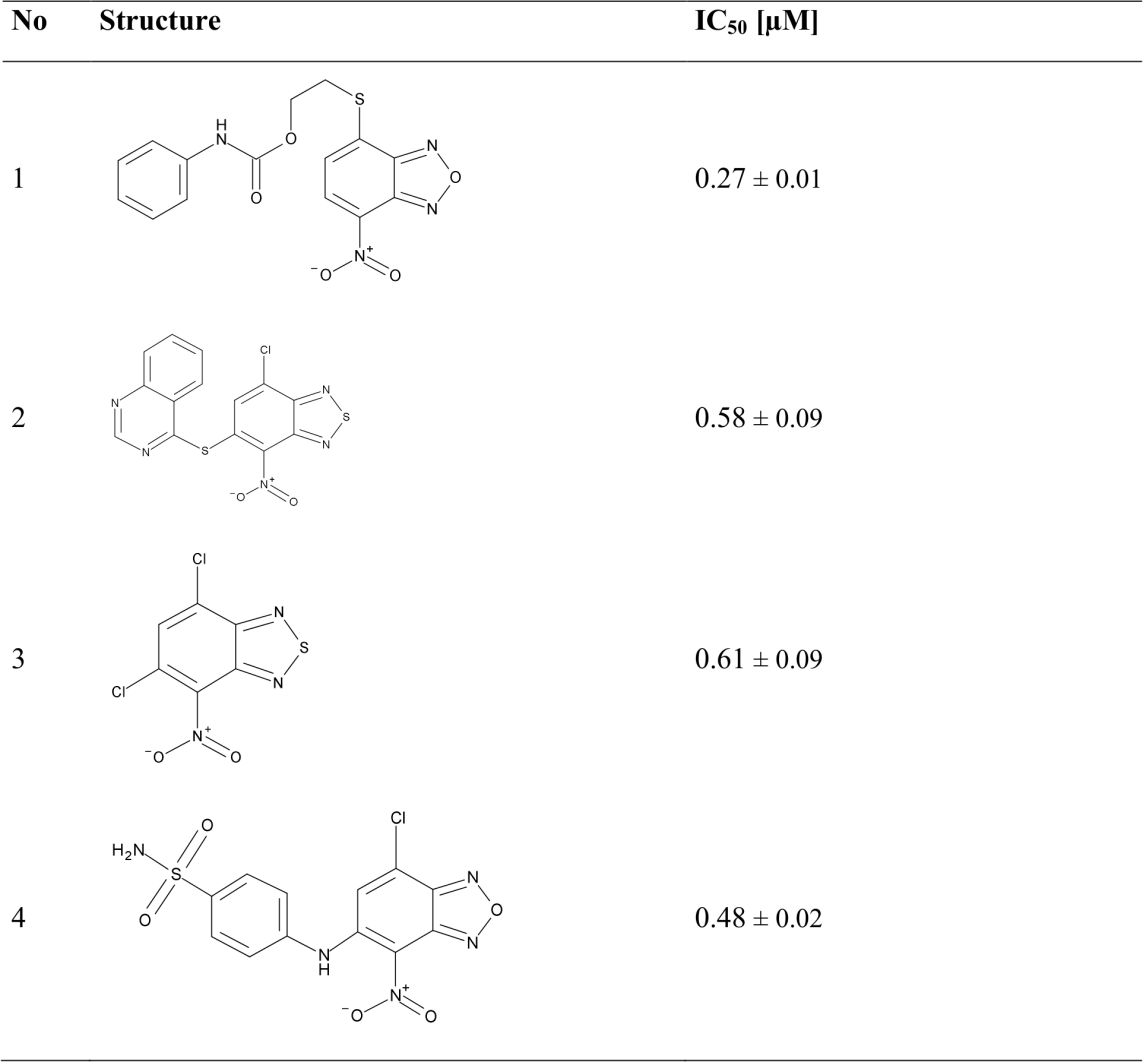
Chemical structures and *in vitro* inhibition data of the four most active compounds. IC50 values have been determined using the same conditions as in the primary assay in the presence of 150 μM TS2. IC50 ± SD (n = 3).

**Fig 7:**
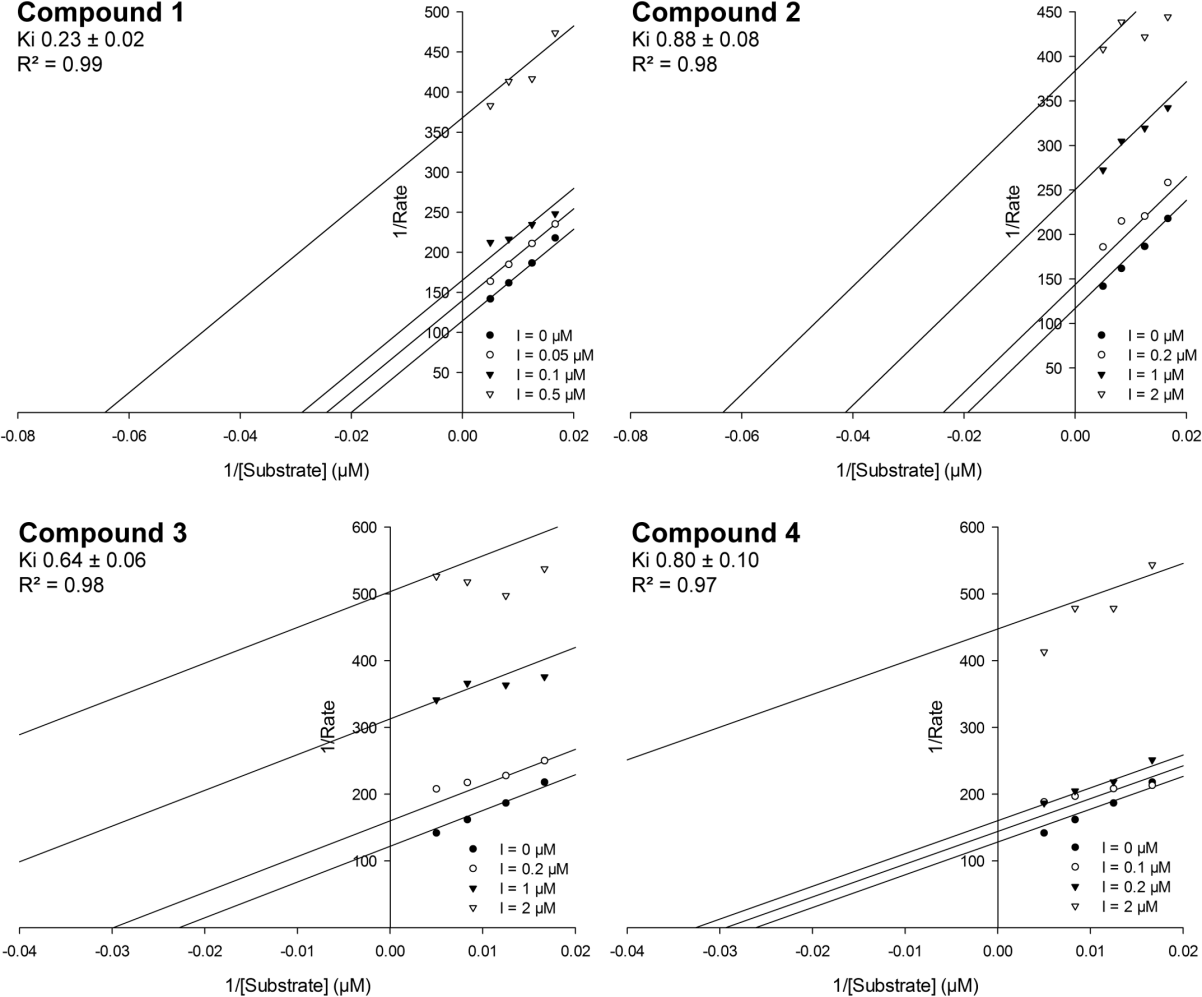
Lineweaver-Burk plots for the most active compounds assuming an uncompetitive mode of inhibition. The K_**i**_ values represent the mean of two independent experiments; standard deviations are based on fitted plots. Data analysis has been performed using the Enzyme Kinetics Module of SigmaPlot, which fits the experimental data to the selected binding model.

Before determining the kinetic constants for the most promising compounds, we investigated whether the compounds bind reversibly or irreversibly to the enzyme. A reaction mixture containing TR, inhibitor and NADPH was incubated for 30 minutes. NADPH reduces the disulfide bridge in the active site and thus allows the putative covalent binding of the inhibitor to an active site cysteine. Afterwards, the protein was separated from the low molecular mass components by centrifugation in an Amicon tube. Inhibitors with a reversible binding mode would be washed out, whereas irreversible binders remain bound to the protein. Finally, the recovered protein solution was subjected to a standard assay to determine the remaining activity. Two controls were run to determine the maximum activity that could be recovered. One contained the protein assay mixture without inhibitor, while the second control was a mixture of TR and the reversible inhibitor chlorhexidine. In both controls, 70–80% activity could be recovered compared to the initial kinetics. The four most promising compounds showing IC_50_ values of < 1 μM (Fig 6) were evaluated. They all revealed a reversible binding mode. Subsequently the inhibitor type and constants were determined. The R^2^ values for the fits in the Lineweaver-Burk plots (Fig 7) for uncompetitive and noncompetitive action were comparable whereas those for competitive inhibition were lower (Table 2). Thus, a competitive inhibition is very unlikely for these compounds. Since the screening assay used high substrate concentrations well above the K_m_ value, it was expected to identify inhibitors which don’t compete with TS_2_ for binding.

**Table 2:** Evaluation of different binding models and corresponding K_i_ values.

	Assumed inhibitor type					
	uncompetitive		noncompetitive		competitive	
	K_i_ [μM]	R^2^	K_i_ [μM]	R^2^	K_i_ [μM]	R^2^
Compound 1	0.23±0.02	0.99	0.33±0.03	0.98	0.06±0.03	0.91
Compound 2	0.88±0.08	0.98	1.3±0.12	0.97	0.26±0.12	0.88
Compound 3	0.64±0.06	0.98	0.91±0.08	0.97	0.15±0.09	0.88
Compound 4	0.80±0.10	0.97	1.11±0.13	0.97	0.19±0.07	0.93

The Enzyme Kinetics Module from SigmaPlot 10 was used to evaluate the inhibition type and K_i_ values. A detailed statistical report was created for each binding model to compare different models and to determine the quality of fit to each model.

### Activity of the compounds versus intact parasites

**Fig 8:**
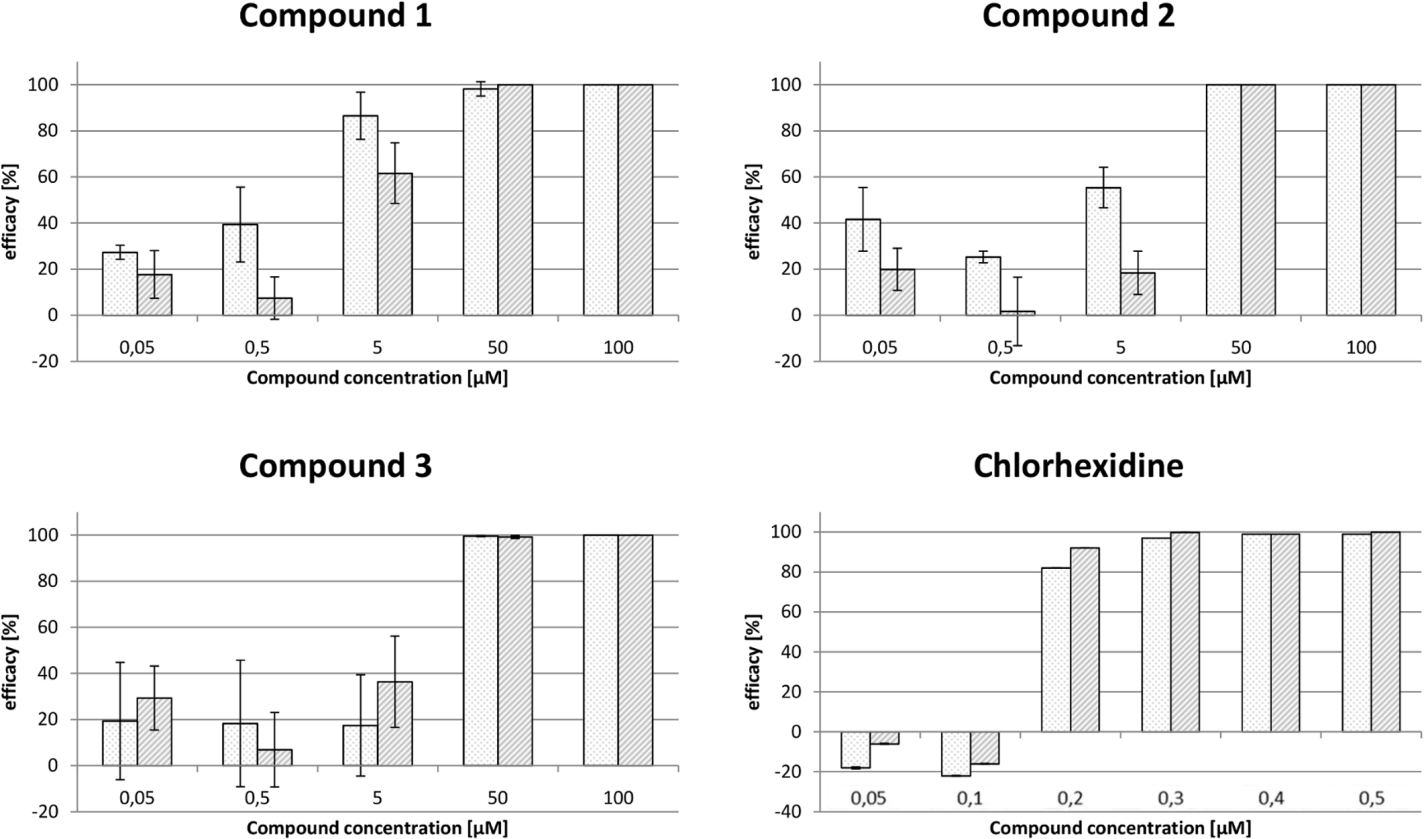
Effect of three top hits on the proliferation of *T*. *brucei*. Bloodstream form trypanosomes were cultured in the presence or absence of the respective compounds. After 48 h (light grey) and 72 h (grey), living cells were counted. The efficacy describes the inhibition of the cell proliferation in the presence of inhibitor compared to DMSO. Chlorhexidine served as positive control. The values are the mean ± SD from three independent series of experiments.

The four most active compounds out of the 82 *in vitro* hits (Fig 6) were tested towards wild-type bloodstream *T*. *brucei*. The parasites were cultured in the presence of different concentrations of the compounds and after 48 h and 72 h living cells were counted. Compounds 1, 2, and 3 inhibited parasite proliferation with EC_50_ values between 50 and 5 μM (Fig 8), whereas compound 4 was not active. Chlorhexidine, which was used as a positive control, displayed an EC_50_ value between 200 and 100 nM. To determine accurate EC_50_ values, compounds 1 and 2 were re-evaluated using the ATPlite 1step assay system (PerkinElmer, Waltham, MA, USA) as described in Füller *et al*.[37]. The assay is based on the emission of light caused by the reaction of ATP with D-luciferin in the presence of luciferase, which is proportional to the amount of ATP serving as a marker for cell viability. The compounds were tested on wild-type cells as well as on parasites transfected with pHD1700-TbTR treated with 1 μg/ml tetracyclin for 1–2 weeks to induce the expression of an ectopic copy of TR. Unfortunately, the *T*. *brucei* strain allowing the down-regulation of TR which was generated more than a decade ago in the laboratory of Christine Clayton [7] is not available anymore. Therefore, this tet-inducible overexpressing system leading to up to three fold higher TR levels had to be used instead. Wild-type cells treated with 1 μg/ml tetracyclin were included to exclude any effect of the antibiotic on parasite proliferation. For compound 1, the ATPlite assay confirmed the time-dependent decrease of inhibition observed in the cell counting experiments, resulting in an EC_50_ value of 2 μM at 24 h and of 15 μM after 72 h (Table 3). Compound 2 displayed also a slightly lower degree of inhibition with time, the EC_50_ value after 72 h being 58 μM compared to 42 μM at 24 h and 48 h. With both inhibitors, no significant difference was observed between wild-type parasites and cells that expressed also the ectopic TR-copy at an approximately 3-fold level compared to wild-type cells. Thus, it could not be proven that the efficacy of these compounds against parasites is really due to inhibition of TR.

**Table 3:** EC_50_-values for bloodstream trypanosomes using the ATPlite assays.

			EC_50_ [μM]	
Time		24 h	48 h	72 h
Compound 1	Wild-type cells	2.1 ± 1.2	4.9 ± 1.3	24.3 ± 2.0
	TR-Over-expressing cells	1.7 ± 1.2	3.4 ± 1.3	17.1 ±1.7
Compound 2	Wild-type cells	56.4 ± 2.0	33.0 ± 2.2	55.9 ± 1.3*
	TR-Over-expressing cells	37.6 ± 1.9	46.9 ± 3.1	82.0 ± 1.6*

All values are the mean ± SD of three series of measurements.

*These 72 h values were calculated from experimental series containing up to 150 μM of the compound corresponding to 1.5% of DMSO which affects cell viability.

In summary, three compounds out of four *in vitro* hits showed activity down to 2 μM in cell culture, which is comparable to the activity of nifurtimox, a nitro-heterocyclic drug against *T*. *cruzi* which is now used in a combination therapy towards African trypanosomes and inhibits proliferation of *T*. *brucei* with an EC_50_ value of 2.5 μM [45].

Compound 4 with the second lowest IC_50_ and K_i_ on the recombinant TR was inactive in bloodstream *T*. *brucei* cell cultures. Inspecting the physicochemical properties of all four compounds revealed that compound 4 is the least lipophilic and has the largest Polar Surface Area (PSA and fractional PSA) indicating poor cell membrane permeability. These initial predictions were confirmed by the ADME permeability parameters CACO2, logBB and SKIN which all predicted a poor permeability for this structure (Table 4). Compound 4 also revealed a very high level for intestinal absorption compared to the other three candidates. These observations support the hypothesis that compound 4 may not enter *T*. *brucei* sufficiently to inactivate TR, assuming that all four compounds are probably incorporated by passive diffusion. The findings are supported by the circumstance that a genome-scale RNA interference target sequencing screen did not identify a transporter for the nitro-aromatic nifurtimox [45] and another nitro-aromatic antitrypanosomal drug megazol enters the parasite by passive diffusion [46]. Surprisingly, chlorhexidine was much more active in the cell culture assays than the best *in vitro* active (compound 1), although the on target activity (K_i_ and IC_50_) of compound 1 was 10–20 times better compared to chlorhexidine. In addition, the predicted membrane permeability of chlorhexidine is even lower compared to compound 4. The poor permeability of chlorhexidine has also been described in many pharmacokinetic studies [47]. All these observations do not explain the observed high activity on bloodstream form trypanosomes of chlorhexidine. It might be that TR is not the only target of chlorhexidine in trypanosomes or that chlorhexidine can enter the parasite e.g. by active transport or interacts with the cell membrane. It is known that the antibacterial activity of chlorhexidine is caused by its incorporation and destabilization of the bacterial cell wall [47]. The time-dependent activity loss of compound 1 and 2 might be caused by stability or solubility issues in the culture medium. Both compounds show the least solubility and metabolic stability values of all four compounds. Nevertheless, such hurdles are rather common in the drug discovery process and can be addressed by further optimization steps.

**Table 4:** Calculated physicochemical properties and ADME parameters.

	Cpd 1	Cpd 2	Cpd 3	Cpd 4	Chlorhexidine
Molecular Weight ^1)^	360.34	375.81	250.06	369.74	505.45
Num Rotatable Bonds ^1)^	7	3	1	4	13
Num H Acceptors ^1)^	7	7	4	7	10
Num H Donors ^1)^	1	0	0	2	6
logP ^2)^	3.547	4.977	3.037	2.686	2.398
Polar Surface Area ^1)^	148.36	150.92	99.84	165.31	177.58
Fractional PSA ^1)^	0.437	0.456	0.477	0.52	0.336
Plasma Protein Binding ^1)^	1	0	1	1	0
Blood Brain Barrier ^1)^	4	4	2	4	4
Intestinal Absorption ^1)^	1	0	0	3	3
ADME Solubility Level ^1)^	2	1	2	2	1
Molecular Solubility ^1)^	-4.817	-5.861	-3.649	-4.599	-8.612
logS 7.5 ^2)^	-4.61	-4.782	-2.865	-4.067	2.497
CACO 2 ^2)^	0.5937	0.861	0.965	0.097	-1.01
logBB ^2)^	-0.479	0.072	0.294	-0.826	-3.013
SKIN ^2)^	-3.068	-2.476	-2.37	-4.964	-7.046
Metabolic Stability ^2)^	45.712	46.826	85.748	70.144	90.223

^1)^ Pipeline Pilot [32]

^2)^ VolSurf+ [36]

### Conclusion

Trypanothione reductase (TR), an essential enzyme and validated target in the redox metabolism of pathogenic trypanosomes, has a large, wide and featureless active site making the protein inappropriate for target-based *in silico* screenings like high-throughput docking. In this paper we presented a new approach to identify novel TR inhibitors as starting points for drug discovery by combining target-based *in vitro* screening and 2D *in silico* screening methods that are capable to exceed the hit identification rates of common pure high-throughput assays or *in silico* approaches. In our screening campaign only ~4.000 compounds had to be tested in the adapted assay to identify 82 novel compounds being more active than chlorhexidine, a well-known TR inhibitor. Four compounds showed TR inhibition in the nanomolar range and three of them also reveal activity against intact bloodstream form *T*. *brucei*. These compounds provide promising starting points for a hit-to-lead process within a drug discovery project, where solubility, stability, toxicity, and activity of derivatives are used to identify the best compounds for further optimization.

The screening compound library and also the diverse subset used in the first target-based *in vitro* screening campaign contained structurally diverse compounds. It was applied as general library in many other target-based screening approaches. Nitro compounds and especially nitroheterocyclic compounds were only represented in a minor extend. Interestingly, with TR as target, this kind of compound class was found with an over-averaged frequency compared to other target-based approaches [48], [49]. Moreover, all best hits including the four compounds studied in detail belong to this specific compound class. Compounds containing nitro groups are usually underrepresented in known drugs. This is related to at least two reasons: a) the nitro group is often not involved in the interactions with the target protein and therefore not essential, b) nitro groups are undesired functional groups that are usually replaced in the lead/drug optimization phase. In contrast, nitroheterocyclic compounds show an over-averaged chemotherapeutical potential in pathogens causing neglected diseases. The interest in this compound class has grown since the success of the nifurtimox/eflornithine combination against African trypanosomiasis and has prompted in many subsequent screening and research activities until today [48], [49]. In addition, the nitro-heterocyclic pro-drug fexinidazole is the first drug candidate in 30 years that entered clinical phase II/III against African trypanosomiasis for both stages of the disease [50], [51]. The compound also shows activity against *T*. *cruzi* and *L*. *donovani* and therefore is evaluated against Chagas disease [50], [52] and Leishmaniasis [53] in clinical proof-of-concept studies. Despite these research efforts the role of the nitro group concerning the activity of such compounds could not be explained yet. *In silico* modeling and analysis of binding modes of compound 1 to TR suggest a potential interaction of the nitro group with Asn339 and/or Arg354 of TR [27]. Although these analyses are artificial to a certain extent, they might give a hint that the nitro group plays an important role in the binding of the compounds to TR. Nevertheless, other explanations like an effect of the nitro group on the specific redox systems of such pathogens are also discussed [48], [48]. Further experiments leading to better understanding of the prominent role of nitro compounds are needed and may push the importance of heterocyclic compounds as therapeutic agents towards neglected diseases.
